# Nitrogen and phosphorus addition differentially enhance seed production of dominant species in a temperate steppe

**DOI:** 10.1002/ece3.8185

**Published:** 2021-10-11

**Authors:** Lei Su, Mengzhou Liu, Chengming You, Qun Guo, Zhongmin Hu, Zhongling Yang, Guoyong Li

**Affiliations:** ^1^ International Joint Research Laboratory for Global Change Ecology School of Life Sciences Henan University Kaifeng China; ^2^ College of Geography and Environmental Science Henan University Kaifeng China; ^3^ Key Laboratory of Geospatial Technology for the Middle and Lower Yellow River Regions (Henan University) Ministry of Education Kaifeng China; ^4^ Key Laboratory of Ecosystem Network Observation and Modeling National Ecosystem Science Data Center Institute of Geographic Sciences and Natural Resources Research Chinese Academy of Sciences Beijing China; ^5^ Forestry Ecological Engineering in the Upper Reaches of the Yangtze River Key Laboratory of Sichuan Province & National Forestry and Grassland Administration Key Laboratory of Forest Resources Conservation and Ecological Safety on the Upper Reaches of the Yangtze River & Rainy Area of West China Plantation Ecosystem Permanent Scientific Research Base Institute of Ecology & Forestry Sichuan Agricultural University Chengdu China; ^6^ School of Geography South China Normal University Guangzhou China; ^7^ Southern Marine Science and Engineering Guangdong Laboratory (Zhuhai) Guangdong China

**Keywords:** nitrogen deposition, nutrient availability, phosphorus enrichment, reproductive allocation, seed number, *Stipa krylovii*

## Abstract

Previous studies have demonstrated changes in plant growth and reproduction in response to nutrient availability, but responses of plant growth and reproduction to multiple levels of nutrient enrichment remain unclear. In this study, a factorial field experiment was performed with manipulation of nitrogen (N) and phosphorus (P) availability to examine seed production of the dominant species, *Stipa krylovii*, in response to N and P addition in a temperate steppe. There were three levels of N and P addition in this experiment, including no N addition (0 g N m^−2^ year^−1^), low N addition (10 g N m^−2^ year^−1^), and high N addition (40 g N m^−2^ year^−1^) for N addition treatment, and no P addition (0 g P m^−2^ year^−1^), low P addition (5 g P m^−2^ year^−1^), and high P addition (10 g P m^−2^ year^−1^) for P addition treatment. Low N addition enhanced seed production by 814%, 1371%, and 1321% under ambient, low, and high P addition levels, respectively. High N addition increased seed production by 2136%, 3560%, and 3550% under ambient, low, and high P addition levels, respectively. However, P addition did not affect seed production in the absence of N addition, but enhanced it under N addition. N addition enhanced seed production mainly by increasing the tiller number and inflorescence abundance per plant, whereas P addition stimulated it by decreasing the plant density yet stimulating height of plants and their seed number per inflorescence. Our results indicate seed production is not limited by P availability but rather by N availability in the temperate steppe, whereas seed production will be increased by P addition when N availability is improved. These findings enable a better understanding of plant reproduction dynamics in the temperate steppe under intensified nutrient enrichment and can inform their improved management in the future.

## Graphical abstract

Phosphorus did not influence seed production in the absence of nitrogen addition. Nitrogen addition enhanced seed production through increasing tiller and inflorescence number. Phosphorus addition stimulated it via stimulating plant height and seed number per inflorescence.

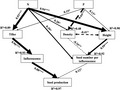

## 1 INTRODUCTION

Anthropogenic‐driven nutrient inputs, represented by nitrogen (N) and phosphorus (P) enrichment in terrestrial ecosystems, have been increasing intensively since the Industrial Revolution (Harpole et al., 2011; Liu, Zuo, et al., 2021; Liu, Zhao, et al., 2021; Phoenix et al., 2012). Global cycles of N and P have been amplified by c.100% and c. 400%, respectively, due to intensified human activities (Elser et al., 2007). Being two crucial nutrient elements for growing plant, N and P enrichment can profoundly influence plant growth, survival, and reproduction, with subsequent impacts on community structure and ecosystem functioning (Long et al., 2020; Zhao, Liu, et al., 2018). Reproduction is an essential function in the life cycles of plants that determine their fitness (Willson, 1983). Seed production is an important index of reproduction that strongly influences the relative ability of species to disperse and establish as seedlings (Liu et al., 2012; Pierce et al., 2014). In addition, seed production can affect the size and extent of soil seed banks and contribute to the maintenance of plant diversity and species composition (Luzuriaga et al., 2005). Therefore, understanding the effects of N and P enrichment upon seed production is critical for predicting plant community structure and, consequently, ecosystem functioning.

Nitrogen is a limiting nutrient for plant reproduction in terrestrial ecosystems. Numerous studies have demonstrated that N enrichment tends to augment seed production in plants (Bogdziewicz et al., 2017; Li, Li, et al., 2016; Ma & Herath, 2016; Shi et al., 2017). For example, in a temperate steppe, *Leymus chinensis* produced more seeds via N enrichment through enhanced spikelet and flower differentiation (Wang et al., 2010). P plays a key role in regulating plant reproductive processes because it can significantly affect the partitioning of assimilation products, flowering phenology, root growth, and seed maturation (Petraglia et al., 2014; Wang et al., 2017). Previous studies have reported that plants under P‐deficient conditions allocate little to reproductive growth, which manifests as a shortened flowering period, reduced seed yield, and weakened dispersal ability (Fujita et al., 2014; Wang et al., 2019). Furthermore, the positive, negative, or neutral effects of P enrichment on seed production can all occur in terrestrial ecosystems (Sims et al., 2012b; Singh et al., 2020; Wang et al., 2017). For example, although P enrichment negligibly affected the seed production of *Stipa kryloii*, it did increase that of *Artemisia frigida* in a temperate steppe (Li et al., 2017).

The availability of N and P, however, may jointly affect seed production since plant growth is predicted to be colimited by multiple resources (Graciano et al., 2006; Harpole & Suding, 2011; Harpole et al., 2016; Long et al., 2016; Peñuelas et al., 2013). Simultaneously adding N and P enhances ecosystem primary productivity much more than adding either of them alone (Elser et al., 2007; Harpole et al., 2011; Solis et al., 2013). By contrast, such an interactive effect between N and P addition was not found for plant reproduction in a temperate steppe (Li et al., 2017). Further, the growth and reproduction of plants may respond differentially to the levels of nutrient enrichment (Bowman et al., 2006; Tang et al., 2017). A meta‐analysis found that plant productivity in meadow steppe is positively related to N addition under low N addition level, but it decreases with increasing N addition under high N addition level (Tang et al., 2017). Nonetheless, few attempts have been made to empirically investigate how N and P addition rates and their interaction could affect plant reproduction allocation and seed production in terrestrial ecosystems, because most nutrient addition studies only include two levels of nutrient treatments (i.e., control vs. nutrient enrichment).

Grassland is one of the major terrestrial ecosystems and covers 40% of the world's land area (Adams et al., 1990). In this respect, the temperate steppe in northern China is representative of the typical vegetation of the Eurasian grassland biome (Bai et al., 2010; Su et al., 2018). A comprehensive project that used three levels of N and P addition was begun in April 2012, aiming to examine the effects of nutrient enrichment on community structure and ecosystem functioning in a typical temperate steppe of Inner Mongolia, northern China. As part of this long‐term project, the present study was done to examine the interactive effects of N and P addition rates upon seed production of the dominant species *Stipa krylovii*, which is the most common perennial grass in typical temperate steppe ecosystems in China. We sought to address the following specific questions: (1) Do changes in N and P availability and in N:P ratio alter the seed production of the dominant species in temperate steppe ecosystem? (2) Which factors determine seed production of the dominant species under the different nutrient addition treatments?

## 2 MATERIALS AND METHODS

### 2.1 Site description

This experimental site is located in a fenced temperate steppe in Duolun County (42°02′N, 116°17′E, 1324 m a.s.l), Inner Mongolia, China. For the years 1960 to 2014, its mean annual temperature was 2.1°C and mean annual precipitation was 371 mm. Soil is classified as chestnut soil according to the Chinese classification, and Calcis‐Orthic Aridisol in the US Soil Taxonomy classification, with sand, silt, and clay contents of 62.8%, 20.3%, and 16.9%, respectively. *Stipa krylovii* is the most common herbaceous species in the temperate steppe; other common species include *Artemisia frigida*, *Agropyron cristatum*, *Cleistogenes squarrosa*, *Allium bidentatum*, and *Potentilla acaulis* (Song et al., 2016; Su et al., 2018).

### 2.2 Experimental design

A randomized block design with complete factorials for N and P addition was used in this experiment, which was set up in early April 2012. Three levels of N addition were crossed with three levels of P addition, producing nine different nutrient addition treatments. For the N addition, the three levels were ambient N (no N added, 0 g N m^−2^ year^−1^), low N addition (10 g N m^−2^ year^−1^), and high N addition (40 g N m^−2^ year^−1^); likewise, for P addition, its three levels were ambient P (no P added, 0 g P m^−2^ year^−1^), low P addition (5 g P m^−2^ year^−1^), and high P addition (10 g P m^−2^ year^−1^). Low N, low P, and high P addition were employed according to the standard nutrient application levels of the “Nutrient Network” (https://nutnet.org/). In our study area, the aboveground biomass reached saturation when N addition was at the rate of 20 g N m^−2^ year^−1^ and then was suppressed at the rate of 30~50 g N m^−2^ year^−1^ (Xu, Liu, et al., 2015). So, the rate of 40 g N m^−2^ year^−1^ was selected as high N addition in this study. The field experiment consisted of four blocks, with each having nine plots randomly assigned to the nine nutrient addition combinations. Each plot was 2 m × 2 m in size, and the distance between any two adjacent plots within each block was 1 m. Nutrient addition was applied to plots annually, in July. Ammonium nitrate (NH_4_NO_3_) and calcium superphosphate (CaH_4_P_2_O_8_) were used in the N and P addition plots, respectively.

### 2.3 Plant sampling

The dominant plant species, *S*. *krylovii*, was chosen to examine the effects of N and P addition upon seed production in this study. Being a widespread perennial tussock grass in typical temperate steppe, it is an important fodder species used in China, Mongolia, Kazakhstan, and Russia (Ronnenberg et al., 2011; Wu & Raven, 2006). The vegetative and reproductive tillers of *S*. *krylovii* can attain heights of 50 cm during the growing season (Li et al., 2017).

In this experiment, ten plant individuals (two or three individuals in each plot) were selected under each treatment to measure tiller number per individual, inflorescence number per individual, seed number per inflorescence, and maximum plant height in late August from 2015 to 2017. Meanwhile, the density of *S*. *krylovii* was investigated in a randomly selected subplot (1 m × 1 m) within each plot. Plant density was calculated by the number of the dominant species divided by subplot area. Seed production per individual was calculated as the product of seed number per inflorescence and inflorescence number per individual (Brys et al., 2005).

### 2.4 Data analysis

Linear mixed effects models were employed to test the main and interactive effects of N addition and P addition upon six response variables: seed production, seed number per inflorescence, inflorescence number, tiller number, density, and maximum height of *S*. *krylovii*. Block was taken as random factor. Significant difference in means for seed production, seed number per inflorescence, inflorescence number, tiller number, density, and maximum height of *S*. *krylovii* among the three levels of N or P addition were compared by Duncan's multiple range test. All the data were log‐transformed to meet homogeneity of variance for linear mixed effects models before these analyses, which were carried out using SAS software (Proc Mixed, SAS 8.1; SAS Institute Inc.). In addition, confirmatory analyses based on structural equation model (SEM) were conducted to quantify the direct and indirect impacts of N and P addition upon seed production. This SEM analysis was carried out in AMOS 21.0 (IBM, SPSS).

## 3 RESULTS

### 3.1 Seed production under the nitrogen and phosphorus addition treatments

**FIGURE 1:**
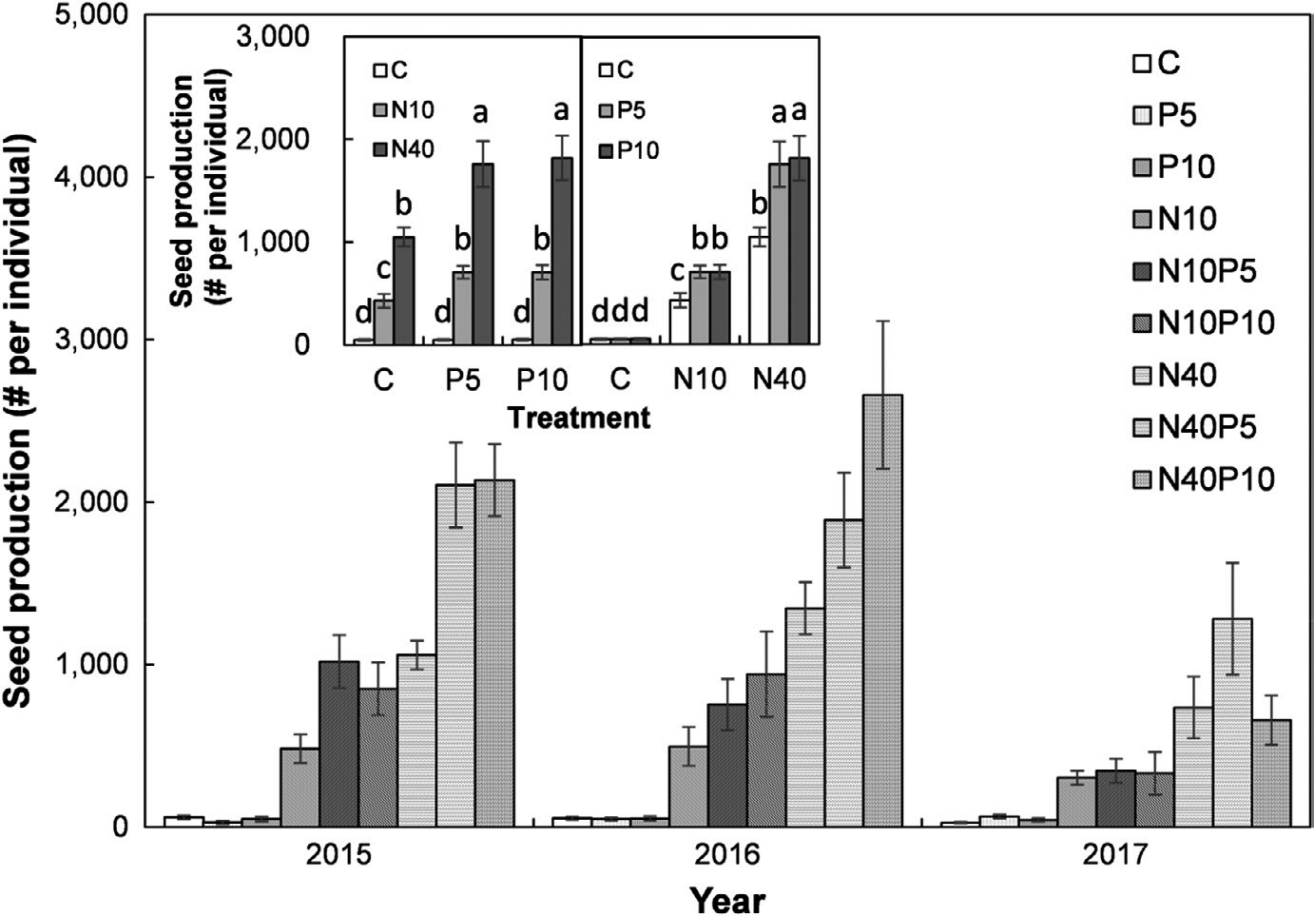
Seed production of *Stipa krylovii* for different treatment combinations of nitrogen (N) and phosphorus (P) addition in a temperate steppe of Inner Mongolia, China. Different letters in the insert panels indicate signiﬁcant differences at *α* level of 0.05. Abbreviations: C, no N and P added; P5, low P addition (5 g P m^−2^ year^−1^); P10, high P addition (10 g P m^−2^ year^−1^); N10, low N addition (10 g N m^−2^ year^−1^); N40, high N addition (40 g N m^−2^ year^−1^)

Across the three sampling years, seed production was consistently and significantly affected by N and P addition in the temperate steppe (both *p* > .05; Figure 1, Table 1). The low N addition enhanced seed production by 814%, 1371%, and 1321% under ambient, low, and high P addition treatments, respectively (all *p* < .05; Figure 1 left insert). The high N addition increased seed production by 2136%, 3560%, and 3550% under ambient, low, and high P addition treatments, respectively (all *p* < .05; Figure 1 left insert). However, neither a low nor a high P addition altered seed production under ambient N (both *p* > .05; Figure 1 right inert). The low and high P addition augmented seed production by 65% and 65% under low N addition, and by 68% and 74% under high N addition treatments, respectively (all *p* < .05; Figure 1 right insert).

**TABLE 1:** Results (*F* ratios) of linear mixed effects models on the effects of N and P addition on the seed production, inflorescence number, seed number per inflorescence, tiller number, density, and height in a temperate steppe of Inner Mongolia, China

	Seed production	Seed number per inflorescence	Inflorescence number	Tiller number	Density	Height
N	108.54***	106.99**	141.38***	160.18***	11.56*	148.13***
P	9.93**	6.86*	3.37	4.11*	35.93***	3.488
N × P	8.00**	1.83	2.54	4.94*	0.76	0.303

Note

Significant level: *p < .05, **p < .01, ***p < .001.

### 3.2 Reproductive traits changed under the nitrogen and phosphorus addition treatments

**FIGURE 2:**
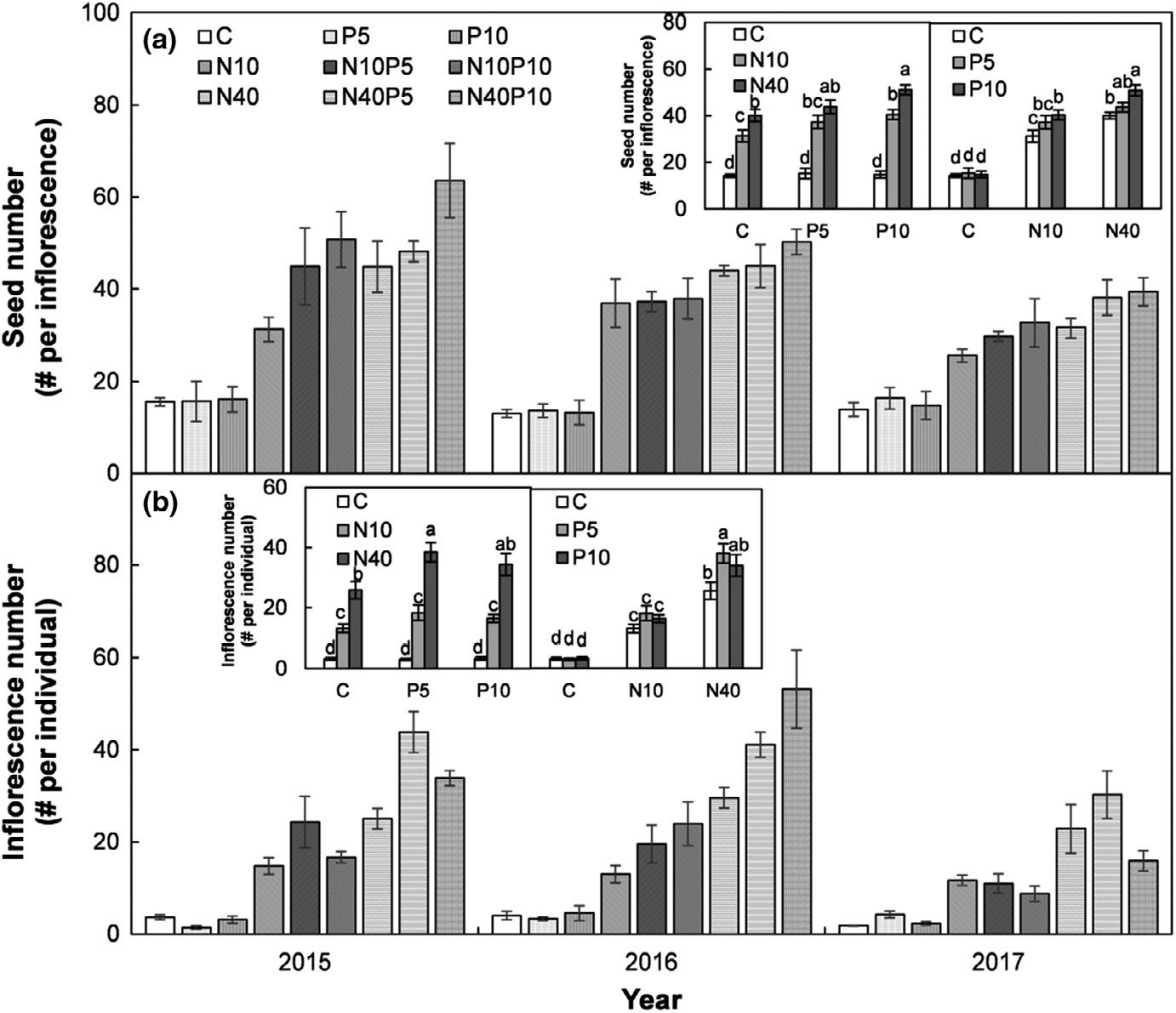
Seed number per inflorescence and inflorescence number of *Stipa krylovii* at different N and P addition treatment levels in a temperate steppe of Inner Mongolia, China. Different letters in the panel indicate significant differences at the *α* level of 0.05. See Figure 1 for the abbreviations

Seed number per inflorescence and inflorescence number were significantly influenced by N addition (both *p* < .001), whereas P addition significantly affected seed number per inflorescence (*p* = .01) but did not influence inflorescence number (*p* > .05; Figure 2, Table 1). The low N addition stimulated seed number per inflorescence by 120%, 145%, and 175% under ambient, low, and high P addition conditions, respectively (all *p* < .05; Figure 2a left insert, Table 1). The high N addition increased seed number per inflorescence by 183%, 187%, and 247% under the ambient, low, and high P addition treatments, respectively (all *p* < .05). Although the low P addition did not affect seed number per inflorescence under ambient, low, or high N addition conditions (all *p* > .05). However, high P addition enhanced seed number per inflorescence by 29% under the low N, and 27% under the high N addition treatments (both *p* < .05), but not under ambient N (*p* > .05; Figure 2a right insert).

The low N addition increased the inflorescence number by 317%, 513%, and 396% under the ambient, low, and high P addition treatments, respectively (all *p* < .05), but this effect was stronger under high N addition with corresponding increases of 719%, 1187%, and 934% under ambient, low, and high P addition conditions, respectively (all *p* < .05; Figure 2b left insert). However, either a low or a high P addition did not influence inflorescence number under the ambient or low N addition treatments (all *p* > .05; Figure 2b right insert). Yet the low P addition was able to significantly augment inflorescence number by 49% under the high N addition treatment (Figure 2b right insert; *p* < .05).

### 3.3 Plant growth in responses to nitrogen and phosphorus addition

**FIGURE 3:**
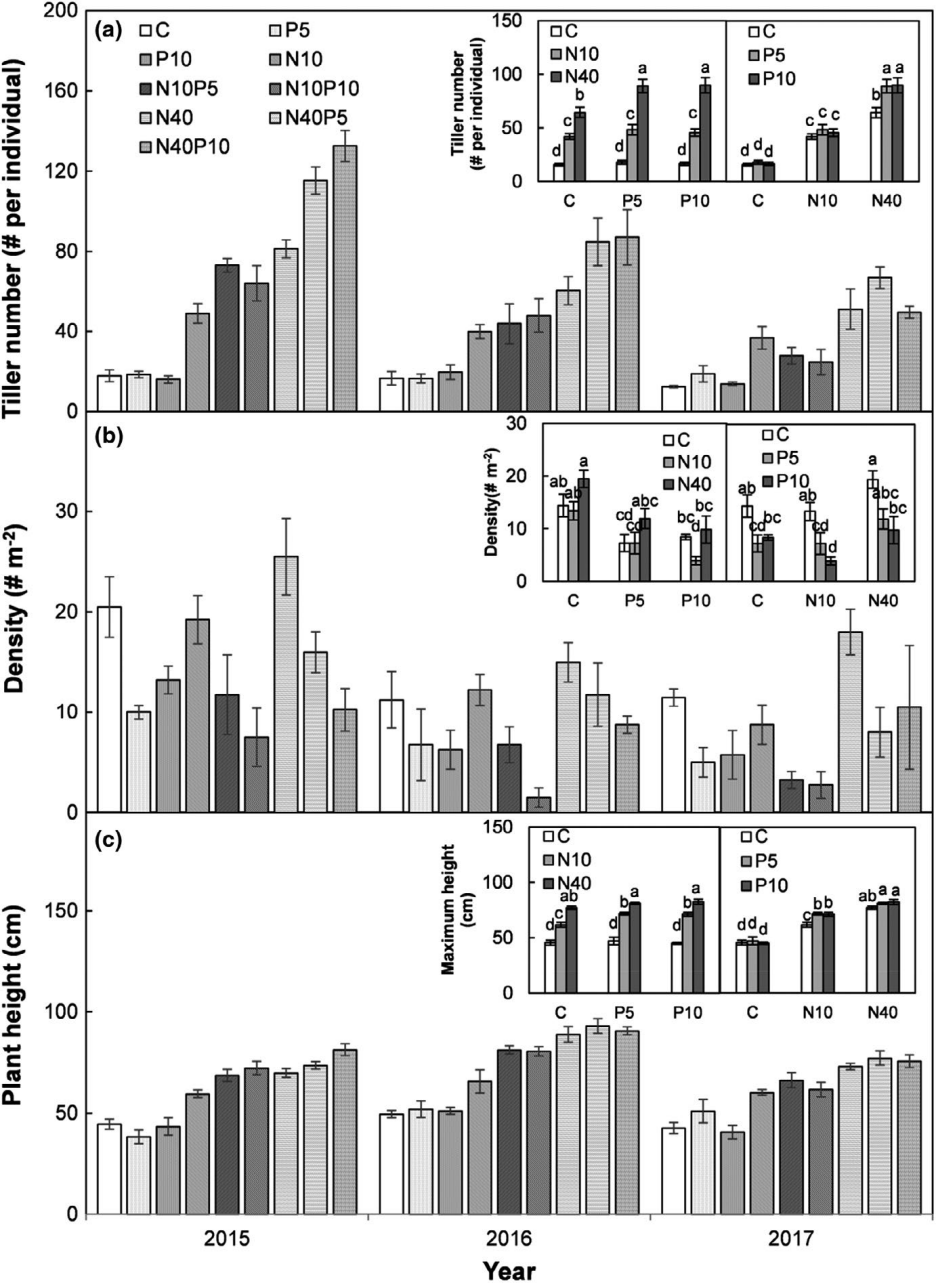
Tiller number, height, and density of *Stipa krylovii* at different N and P addition treatment levels in a temperate steppe of Inner Mongolia, China. Different letters in the panel indicate significant differences at *α* level 0.05. See Figure 1 for the abbreviations

Tiller number, plant density, and maximum plant height were significantly affected by N and P addition across the three surveyed years (all *p* < .05; Figure 3, Table 1). The low N addition increased the tiller number by 168%, 170%, and 177% under the ambient, low, and high P addition treatments, respectively (all *p* < .05; Figure 3a left insert), as did the high N addition, but almost twice as strongly, with corresponding percentages of 313%, 397%, and 446% under ambient, low, and high P addition conditions, respectively (all *p* < .05; Figure 3a left insert). Although the low and high P addition negligibly affected tiller number under the ambient or low N addition treatments (all *p* > .05; Figure 3a right insert), they did raise tiller number by 38 and 40% under the high N addition treatments, respectively (both *p* < .05; Figure 3a right insert).

The low N addition did not influence the density of *S*. *kryloii* under ambient and low P addition treatments (both *p* > .05), but suppressed it by 53% under high P addition (*p* < .05; Figure 3b left insert). By contrast, high N addition enhanced plant density by 35% and 64% under ambient and low P addition, respectively (both *p* < .05), but did not affect it under high P addition (*p* > .05; Figure 3b left insert). Across the three years, low P addition decreased the density, on average, by 49% under ambient N conditions (*p* < .05). While low and high P addition, respectively, reduced plant density by 46% and 71% under low N addition (both *p* < .05), the effect of high P addition was weakened by the high N addition, so that plant density decreased by 50% (*p* < .05; Figure 3b right insert).

The low N addition enhanced the maximum plant height by 36%, 52%, and 58% under ambient, low, and high P addition treatments, respectively (all *p* < .05), but the corresponding effects were stronger, at 69%, 72%, and 83% for the high N addition (Figure 3c left insert; all *p* < .05). Although the low and high P addition did not influence the maximum height under ambient or high N addition conditions (Figure 3c right insert; all *p* > .05), they did so under the low N addition treatments by 16% and 15%, respectively (Figure 3c right insert; *p* < .05).

### 3.4 Path analysis for effects of reproductive traits and plant growth on seed production

**FIGURE 4:**
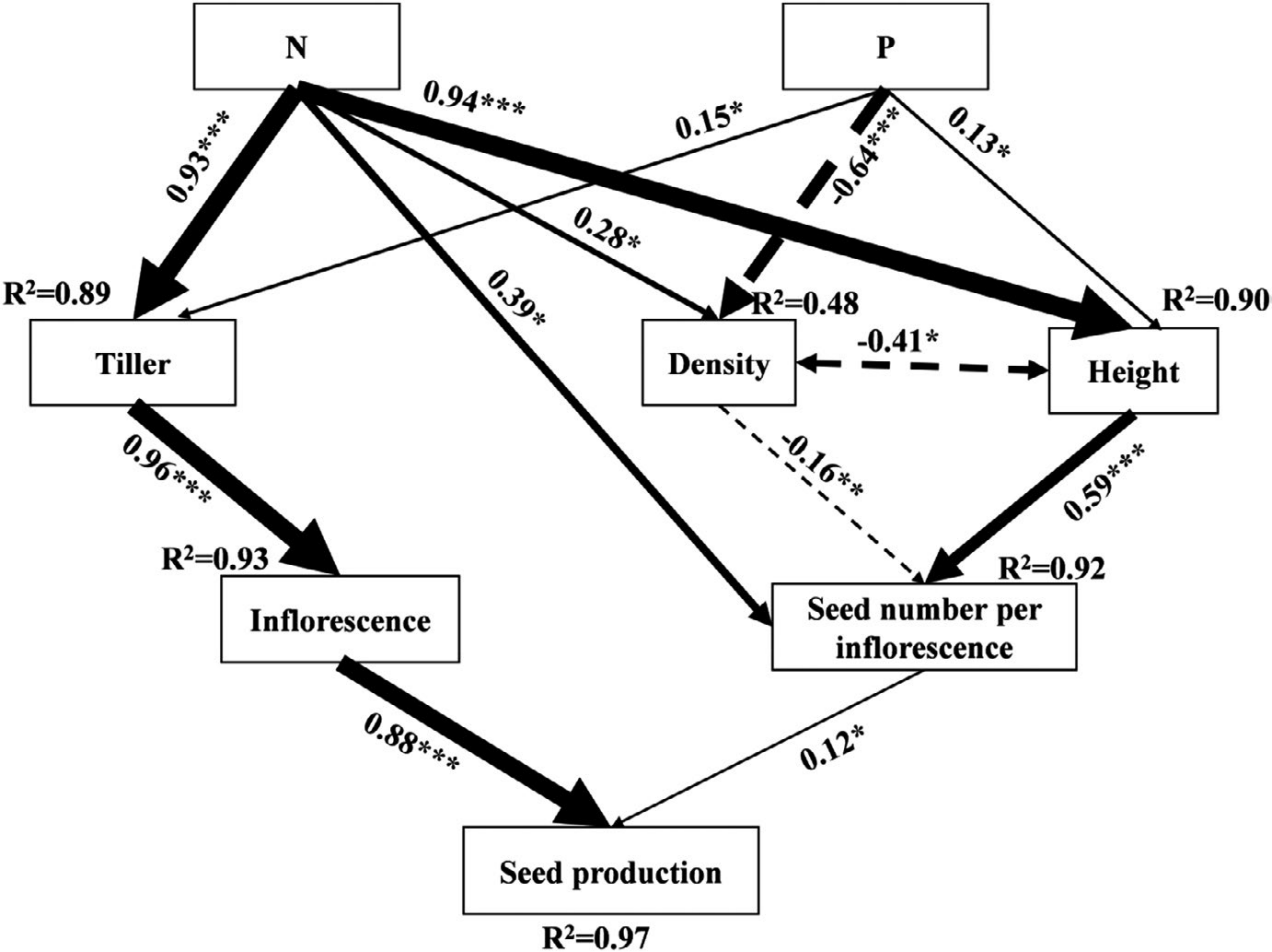
Structural equation model (SEM) analysis of causal relationships of seed production to changes in tiller number, plant density, plant height, seed number per inflorescence, and inflorescence number of *Stipa krylovii* in a temperate steppe of Inner Mongolia, China. All arrows represent significant relationships (*p* < .05). Results of the model fitting: *χ* ^2^ = 24.47, *p* = .058, df = 15, RMSEA = 0.134 (note that high *p‐*values associated with *χ* ^2^ tests indicate good model fit to the data). The *R* ^2^ values associated with response variables indicate the proportion of variation explained by relationships with other variables. Dashed and solid arrows indicate negative and positive effects, respectively. The strength of a given relationship is indicated by the width of its arrow

We used SEM to examine the direct and indirect factors affecting seed production. The results revealed that tiller number, plant density, and maximum plant height were indirectly responsible for 89%, 48%, and 90% of the variation in seed production, respectively, under the N and P addition treatments (*χ* ^2^_15_ = 24.47, *p* = .058, RMSEA = 0.134; Figure 4). Direct contributions to changes in seed production arose from the seed number per inflorescence (*R* ^2^ = .88, *p* < .001) and inflorescence number (*R* ^2^ = .12, *p* = .044). The N addition promoted seed production of *S*. *kryloii* mainly via enhanced tiller number and an accompanying enhancement in the plants’ inflorescence number (Figure 4). The P addition increased seed production differently, mainly by reducing the density of *S*. *kryloii*, thereby enabling individuals to gain a greater maximum height and consequently a greater seed number per inflorescence (Figure 4).

## 4 DISCUSSION

### 4.1 Nitrogen addition impact on seed production

The general positive effects of N addition on the seed production of *Stipa krylovii* and this plant's greater seed production under high N addition relative to low N addition together suggest that seed production of this dominant species’ fecundity is limited by N availability in the temperate steppe. This enhanced seed production under N addition is consistent with previous field studies done in grasslands (HilleRisLambers et al., 2009; Li et al., 2017; Shi et al., 2017). Adding N addition to soil can stimulate the plants’ development of its progeny via increased reproductive traits and qualities, such as a greater inflorescence number, or seed number per inflorescence (HilleRisLambers et al., 2009; Shi et al., 2017), and by raising the nutrient concentration of structures for production (DiManno & Ostertag, 2016). However, some researchers have reported that N addition is irrelevant for seed production (DiManno & Ostertag, 2016; Ostertag, 2010). Some plant species may produce plenty of flower nectar, this being rich in amino acids and acting as N storage pool for fruits, leaves, or roots development, which would greatly weaken the promoted allocation of N to seed formation (DiManno & Ostertag, 2016). In our studied steppe grassland, *S*. *krylovii* cannot develop flower nectar, which permits the allocation of nutrients to vegetative or reproductive growth rather than storing them.

Nitrogen addition can promote biomass accumulation via enhanced photosynthesis (Domingues et al., 2015), roots’ extension and expansion (Ruffel et al., 2011), and plant growth (Sims et al., 2012a). In our study, we found that tiller number, plant density, and plant height all increased in N addition conditions, which agrees with other studies that showed that N enrichment enhances plant productivity (Tang et al., 2017; Xu, Fang, et al., 2015). Along with N‐induced vegetative growth, plants usually allocate proportionally more resources to reproductive structures (Liu, Zuo, et al., 2021; Liu, Zhao, et al., 2021; Willis & Hulme, 2004; Xia & Wan, 2013), leading to more reproductive tillers and less abortion of flowers and fruits (Marcelis et al., 2004; Stephenson, 1981). In this study, we found that most tillers in N addition plots attained a high reproductive capacity, which greatly enhanced their inflorescence number and thereby contributed substantially to boosting seed production per individual (Figure 4).

### 4.2 Phosphorus addition impact on seed production

In our study, although the main effect of phosphorus upon seed production of *S*. *krylovii* was significant, neither low nor high P addition influenced seed production in the absence of N addition, suggesting that seed production is not limited by P availability in the temperate steppe. This finding is consistent with previous research (Li et al., 2017; Yang et al., 2014). Both low and high P addition increased seed production in the presence of N addition, and the increment was significantly higher under high N addition than under low N addition, indicating that a P limitation of seed production can be triggered by N addition. This phenomenon is supported by model simulation work (Menge & Field, 2007) as well several field experiments (Marklein & Houlton, 2012; Zheng et al., 2018). Plants capable of a high growth rate under N‐rich conditions will require a greater allocation of P‐rich rRNA to support macromolecular (protein, rRNA) synthesis (Niklas et al., 2005). The demand for P increases with N addition‐induced growth (Li, Niu, et al., 2016). Accordingly, fertilization with P would allow for an increased allocation of P to reproduction.

Phosphorus is not only a structural element of cell organelles (such as mitochondria and chloroplast) but also the primary constituent of phospholipids (ATP and NADPH) that are used for energy metabolism in light and dark reactions. Indeed, P is indispensable for plant photosynthesis and respiration, such that changes in the P concentration available for plant uptake would alter their vegetative and reproductive growth (George et al., 2016; Patel et al., 2017). An external P addition usually tends to enhance plants’ internal P concentration, accelerating their photosynthetic efficiency and thus promoting biomass accumulation (Graciano et al., 2006; Suriyagoda et al., 2014). P enrichment can indirectly promote plant height growth and thereby augment seed number per inflorescence (Figure 4). Higher levels of P to plants can result in more spikelets per fertile tiller (Wang et al., 2017) and an earlier plant flowering date (Petraglia et al., 2014). Both outcomes may subsequently enhance overall fecundity and prolong the seed development period and eventually stimulate seed production.

In addition, soil P availability is highly responsive to local available N (Marklein & Houlton, 2012). Even a minor increase in available N addition can increase soil P availability by stimulating greater root surface phosphatase activity and facilitating P dissolution, which alleviates P limitation (Crowley et al., 2012; Johnson et al., 1999; Schleuss et al., 2020). Although N fertilizer can promote P cycling, the increased available P is insufficient to balance the greater plant demand for P (Li, Niu, et al., 2016); hence, P limitation will gradually become predominant (Peng et al., 2017; Peñuelas et al., 2013).

### 4.3 Implication for fertilization management

Although numerous studies have found that N and P interact to control plant growth, nutrient absorption, and reproductive allocation under conditions of N and P addition (Li et al., 2017; Long et al., 2016; Zhao, Yang, et al., 2018), the effect of multilevel N/P addition on plant reproduction is still unclear. In our study, the effects of N addition upon seed production did not differ significantly under the low or high P addition. A low P addition is sufficient to balance the increased P demand of plant growth, while a high P addition cannot be fully utilized by plants, leading to a similar effect arising between these two levels of P addition. The leaf N:P ratio tends to balance out at a soil available N:P supply ratio of approximately 20 (Zhan et al., 2017). Thus, our low rate of P addition (5 g m^−2^ year^−1^) may need 100 g N m^−2^ year^−1^ to balance the N demand from plant growth in this temperate steppe.

Our study demonstrates the importance of N and P enrichment in regulating the seed production of dominant species in a temperate steppe. Seed production in response to changing available nutrients in soil can profoundly determine plant community structure and dynamics (Basto et al., 2015). Our findings of increased seed production of the dominant species under both N and P addition treatments, coupled to their additive effects, suggest that the *S*. *krylovii* will become more dominant under accelerating N and P enrichment regimes.

Nutrient enrichment has an obvious promoting effect on the seed production of dominant species in natural ecosystems, which provides new insight into the mechanisms of biodiversity loss in the context of intensifying human activities in grasslands especially. Besides, the seed viability, which determines seed survival chance and plant performance, would greatly affect the number and competitiveness of the species in a community. If we want to precisely predict the dynamics of plant community, the seed viability should be taken as an important supplementary indicator for seed production. Moreover, it is confirmed that nutrient enrichment makes the dominant species more productive, implying higher probability of colonization. It could be expected that the dominance of *S*. *kryloii* would be enhanced in the temperate steppe under intensified nutrient enrichment in the future.

## 5 CONCLUSIONS

Seed production of the dominant species in the temperate steppe was enhanced by N addition, and a high level of N addition stimulated seed production more than a low N addition. Whereas seed production was unchanged by P addition alone, it was increased when the latter was in the presence of N addition. Seed production was enhanced mainly through an increasing of tiller and inflorescence numbers under N addition and by decreased plant density stimulating plant height growth and enabling seed number per inflorescence under P addition. Our results indicate that N availability is the main factor limiting seed production, but seed production can become limited by P availability as N enrichment increases in the temperate steppe. These timely findings can facilitate better understanding of grassland seed banks and plant community structure responses to simultaneous multiple nutrient enrichment under future nutrient enrichment scenarios.

## CONFLICT OF INTEREST

The authors declare no conflict of interest.

## AUTHOR CONTRIBUTIONS

**Lei Su:** Data curation (lead); Formal analysis (lead); Writing‐original draft (equal). **Mengzhou Liu:** Formal analysis (lead); Writing‐original draft (equal). **Chengming You:** Resources (equal); Software (equal); Supervision (equal); Validation (equal). **Qun Guo:** Resources (equal); Software (equal); Supervision (equal); Validation (equal). **Zhongmin Hu:** Resources (equal); Software (equal); Supervision (equal); Validation (equal). **Zhongling Yang:** Data curation (lead); Investigation (lead); Project administration (lead). **Guoyong Li:** Conceptualization (lead); Visualization (supporting); Writing‐review and editing (lead).

## Data Availability

The data of this study are available in Dryad. https://doi.org/10.5061/dryad.8sf7m0cms.
